# Genotypic and Lipid Analyses of Strains From the Archaeal Genus *Halorubrum* Reveal Insights Into Their Taxonomy, Divergence, and Population Structure

**DOI:** 10.3389/fmicb.2018.00512

**Published:** 2018-03-29

**Authors:** Rafael R. de la Haba, Paulina Corral, Cristina Sánchez-Porro, Carmen Infante-Domínguez, Andrea M. Makkay, Mohammad A. Amoozegar, Antonio Ventosa, R. Thane Papke

**Affiliations:** ^1^Department of Microbiology and Parasitology, Faculty of Pharmacy, University of Sevilla, Sevilla, Spain; ^2^Department of Molecular and Cell Biology, University of Connecticut, Storrs, CT, United States; ^3^Department of Microbiology, Faculty of Biology and Center of Excellence in Phylogeny of Living Organisms, College of Science, University of Tehran, Tehran, Iran

**Keywords:** *Halorubrum*, taxonomy, divergence, MLSA, polar lipid, ANI, DNA–DNA hybridization

## Abstract

To gain a better understanding of how divergence occurs, and how taxonomy can benefit from studying natural populations, we isolated and examined 25 closely related *Halorubrum* strains obtained from different hypersaline communities and compared them to validly named species and other reference strains using five taxonomic study approaches: phylogenetic analysis using the 16S rRNA gene and multilocus sequencing analysis (MLSA), polar lipid profiles (PLP), average nucleotide identity (ANI) and DNA-DNA hybridization (DDH). 16S rRNA gene sequence could not differentiate the newly isolated strains from described species, while MLSA grouped strains into three major clusters. Two of those MLSA clusters distinguished candidates for new species. The third cluster with concatenated sequence identity equal to or greater than 97.5% was comprised of strains from Aran-Bidgol Lake (Iran) and solar salterns in Namibia and Spain, and two previously described species isolated from Mexico and Algeria. PLP and DDH analyses showed that Aran-Bidgol strains formed uniform populations, and that strains isolated from other geographic locations were heterogeneous and divergent, indicating that they may constitute different species. Therefore, applying only sequencing approaches and similarity cutoffs for circumscribing species may be too conservative, lumping concealed diversity into a single taxon. Further, our data support the interpretation that local populations experience unique evolutionary homogenization pressures, and once relieved of insular constraints (e.g., through migration) are free to diverge.

## Introduction

The genus *Halorubrum* was proposed in order to accommodate the species *Halobacterium saccharovorum, Halobacterium sodomense, Halobacterium trapanicum*, and *Halobacterium lacusprofundi* (McGenity and Grant, 1995). Currently, it is the largest genus in the class *Halobacteria*, and was recently assigned to the newly described family *Halorubraceae* within the order *Haloferacales* (Domain Archaea) (Gupta et al., 2016), and at present contains 36 species with validly published names (Parte, 2018). Members of this genus are phenotypically diverse but all are metabolically aerobic, chemoorganotrophic, and obligately halophilic with growth occurring in media containing 1.0–5.2 M NaCl. They have been isolated from marine salterns, salt lakes, coastal sabkhas, hypersaline soda lakes, saline soils and salt-fermented seafood (Oren et al., 2009; Amoozegar et al., 2017) and they are frequently the numerically dominant microorganisms present in hypersaline environments, as revealed through both culture-dependent and culture-independent techniques (Ghai et al., 2011; Makhdoumi-Kakhki et al., 2012; Ma and Gong, 2013; Fernández et al., 2014a,b; Ventosa et al., 2015).

The 16S rRNA gene sequence is a universal phylogenetic marker within the prokaryotes, and therefore considered essential in taxonomic studies of haloarchaea, including the genus *Halorubrum*. However, over the years it has been demonstrated that the 16S rRNA gene has many disadvantages to its utilization as a phylogenetic and taxonomic marker for the class *Halobacteria* (also known as the haloarchaea). Its highly conserved nature does not allow relevant discernable differentiation among closely related species (for example, 99.4% sequence similarity between *Halorubrum californiense* and *Halorubrum chaoviator* [Pesenti et al., 2008; Mancinelli et al., 2009]); the rRNA operons experience intragenic recombination (Boucher et al., 2004), resulting in reticulated evolutionary histories. It was the most frequently transferred gene among closely related but otherwise distinct lineages (Papke, 2009), making haloarchaeal phylogeny and taxonomy difficult to interpret. Additionally, many haloarchaeal genera have multiple divergent copies of rRNA genes with greater than 6% sequence dissimilarity -the divergence between copies in a single cell can be equal to that seen between genera- have been described within the class *Halobacteria* (Boucher et al., 2004; Sun et al., 2013). To overcome some of these limitations, protein-encoding housekeeping genes have been suggested as alternative phylogenetic markers within the class *Halobacteria* such as *radA* or *rpoB*' genes (Sandler et al., 1999; Walsh et al., 2004; Enache et al., 2007; Minegishi et al., 2010). However, because haloarchaea are known for their frequent recombination across great genetic distances (Nelson-Sathi et al., 2012; Williams et al., 2012; DeMaere et al., 2013) reliance on a single genetic marker cannot provide enough useful phylogenetic information and may lead to taxonomic confusion. Therefore, a multilocus sequence analysis (MLSA) approach that excludes using the 16S rRNA gene was proposed as a preferred methodology for classification and evolutionary studies of the *Halobacteria* (Papke et al., 2011). More recently, a set of five housekeeping genes, i.e., *atpB, EF-2, glnA, ppsA*, and *rpoB'*, have been suggested as recommended markers for the genus *Halorubrum* (Fullmer et al., 2014; Ram Mohan et al., 2014). The use of this MLSA approach instead of the employment of 16S rRNA gene sequence analysis has been endorsed by the ICSP-Subcommittee on the taxonomy of *Halobacteria* (Oren and Ventosa, 2013, 2016).

The protocol for species taxonomy of prokaryotes ultimately relies on DNA-DNA hybridization (DDH) as the “gold standard” for defining bacterial species (Wayne et al., 1987; Stackebrandt et al., 2002). As with 16S rRNA gene sequencing, it too has many associated issues discussed elsewhere (e.g., Johnson, 1994; Zeigler, 2003; Hanage et al., 2006). The use of MLSA as an alternative method for species demarcation in prokaryotes has been successfully applied to several bacterial groups, e.g., lactic acid bacteria (Naser et al., 2006), *Borrelia* (Richter et al., 2006), mycobacteria (Mignard and Flandrois, 2008), pseudomonads and relatives (Young and Park, 2007), *Ensifer* (Martens et al., 2008), *Vibrionaceae* (Pascual et al., 2010; López-Hermoso et al., 2017), and *Aeromonas* (Martinez-Murcia et al., 2011; Roger et al., 2012). Though clearly useful for phylogeny, the degree of congruence between the MLSA and DNA-DNA reassociation data has not been established for taxonomic purposes within the haloarchaea. Therefore, one focus of this study was to compare the MLSA results with DDH and other polyphasic analyses for the genus *Halorubrum* specifically, but also the haloarchaea in general. Polar lipid analysis, a powerful taxonomic marker in haloarchaea (Torreblanca et al., 1986; Oren et al., 2009), and average nucleotide identity (ANI), which has advanced the understanding of prokaryotic taxonomy in other taxa (Konstantinidis and Tiedje, 2005; Goris et al., 2007; Chun and Rainey, 2014; Rosselló-Móra and Amann, 2015; Chun et al., 2018) were compared in this study to assess their usefulness in capturing haloarchaeal taxonomy, divergence and population structure.

## Materials and methods

### Strains and culture conditions

Most of the *Halorubrum* strains used in this study, and other strains from members of the class *Halobacteria* used for comparative analysis, were obtained from the respective culture collection (Table S1) and cultivated following the media and growth conditions recommended by the culture collections. Several strains were isolated in this study from sediment samples of the hypersaline lake Aran-Bidgol, Iran (Table S1), using the plate dilution technique on YPC (Yeast extract, Peptone, Casamino acids) medium, which contained a mixture of 20% (w/v) salts (15.0% NaCl, 2.3% MgSO_4_, 2% MgCl_2_, 0.6% KCl, 0.01% MnCl_2_), 10 mM Tris-HCl (pH 8), 0.5% yeast extract, 1% peptone, and 1% casamino acids, after incubation at 37°C for 15 days. The remaining strains isolated in this study were recovered from hypersaline water samples of a solar saltern in the Namibia desert and salterns in Huelva, Spain (Table S1), again using the plate dilution technique on Halophilic Medium (HM) (Ventosa et al., 1982) with ~20% (w/v) total salts (17.8% NaCl, 0.1% MgSO_4_, 0.036% CaCl_2_, 0.2% KCl, 0.006% NaHCO_3_, 0.023% NaBr, traces of FeCl_3_), 1% yeast extract (Difco), 0.5% proteose-peptone no. 3 (Difco), and 0.1% glucose. The pH was adjusted to 7.2 with 1 M KOH. For routine growth the strains were cultivated in modified SW20 (SeaWater 20%) medium (Rodríguez-Valera et al., 1980) with 20% (w/v) total salts (16.2% NaCl, 1.4% MgCl_2_, 1.92% MgSO_4_, 0.072% CaCl_2_, 0.4% KCl, 0.012% NaHCO_3_, 0.0052% NaBr), 0.5% yeast extract (Difco), and 0.5% casamino acids. The pH was adjusted to 7.2 with 1 M KOH. When necessary, solid media were prepared by adding 2.0% (w/v) Bacto-agar (Difco). These cultures were maintained at−80°C as suspensions (prepared with 15%, v/v glycerol) in modified SW20 medium.

### DNA preparation

Genomic DNA from each culture was isolated and purified using standard methods (Qiagen kit), quantified and checked for quality using a Nanodrop spectrophotometer ND-1000 at 260/280 nm and diluted with TE (10 mM Tris, pH 8.0, 1 mM EDTA) to 20 ng μl^−1^ for subsequent PCR analysis.

### Amplification and sequencing

From each strain, the following five genes were amplified and sequenced: *atpB* (ATP synthase subunit B), *EF-2* (elongation factor 2), *glnA* (glutamine synthetase), *ppsA* (phosphoenolpyruvate synthase), and *rpoB*' (RNA polymerase subunit B'). These genes were chosen for analysis because they are single copy protein-encoding genes previously investigated with success in haloarchaea (Fullmer et al., 2014; Ram Mohan et al., 2014) and recently recommended for MLSA scheme by the ICSP-Subcommittee on the taxonomy of *Halobacteria* (Oren and Ventosa, 2013, 2016). Primers used for amplification and sequencing (Papke et al., 2011; Fullmer et al., 2014) annealed the respective locus across the *Halobacteria*, and therefore one to three degenerated positions were included into the primers, with the exception of rpoB_962F_M13 primer. Additionally, to enhance the sequencing success rate primers containing M13 sequence were used as reported elsewhere (Fullmer et al., 2014; Ram Mohan et al., 2014) (Table S2). The 16S rRNA gene was amplified and sequenced for those strains isolated from the lake Aran-Bidgol and from the salterns in Namibia and Spain, using previously described universal primers (Arahal et al., 1996; López-García et al., 2001) (Table S2).

PCR amplification was performed in a 50 μl reaction mixture composed of 5.0 μl 10 × PCR buffer, 1.5 μl MgCl_2_ (50 mM stock), 1.0 μl dNTPs (10 mM each), 2.0 μl each forward and reverse primers (10 μM), 1.0 μl *Taq* polymerase (5 U μl^−1^; Invitrogen *Taq* DNA Polymerase Native or Roche Fast Start Universal SYBR Green Master [Rox]), 1.0 μl template DNA (20 ng μl^−1^), and ddH_2_O to a final volume of 50 μl. All reactions were performed in an Eppendorf Mastercycler Ep gradient thermocycler (Eppendorf). The PCR cycling conditions included an initial denaturation step (1 min, 94°C) followed by 30 cycles of denaturation (1 min, 94°C), annealing (1 min) and extension (1 min, 72°C) and a final extension period (5 min, 72°C). The annealing temperature for the thermal profile was optimized for each primer set and is shown in Table S2.

The PCR amplicons were examined by agarose gel electrophoresis (1%) and stained with ethidium bromide. Purification of the amplicons was carried out by using standard procedures and sequenced in both directions by the dideoxynucleotide chain termination method using the BigDye chemistry on an ABI 3130XL DNA Analyzer or an ABI 3730XL DNA Analyzer (Applied Biosystems), according to the manufacturer's instructions. Sequences belonging to the same locus were assembled using the software package Geneious (http://www.geneious.com/) and edited manually to resolve ambiguous positions. For several strains with sequenced genomes, housekeeping gene sequences were retrieved from GenBank/EMBL/DDBJ databases. In the case of *Halorubrum californiense* DSM 19288^T^, it was not possible to obtain the *atpB* gene sequence probably because its chromosome has not been completely assembled: the possibility that this strain does not require *atpB* for survival in nature is exceedingly remote. Several 16S rRNA gene sequences were also retrieved from the GenBank/EMBL/DDBJ databases (Table S1).

In summary, five protein-encoding genes from 55 strains (30 type strains and 25 new isolates) within the genus *Halorubrum* were obtained and analyzed. Unfortunately, for *glnA* and *ppsA* genes there were one and two isolated strains, respectively, from which the sequence could not be obtained, no matter how many attempts were made or conditions were tested (Table S1). Gene sequences of *Haloarcula vallismortis* ATCC 29715^T^, *Halobacterium salinarum* R1^T^, and *Haloferax volcanii* DS2^T^/NCIMB 2287^T^ were also included for phylogenetic analysis as outgroups.

### Multiple sequence alignments

DNA sequences for each housekeeping gene were aligned using Muscle version 3.6 (Edgar, 2004a,b) taking into account the corresponding amino acid alignments for protein-coding genes. Alignments were edited using Mesquite version 2.75 (Maddison and Maddison, 2011). Individual gene alignments were concatenated in the following order: *atpB, EF-2, glnA, ppsA* and *rpoB'*. For the 16S rRNA gene, the obtained sequences were aligned using the ARB software (Ludwig et al., 2004).

The analyzed lengths of sequence data determined from the multiple alignments were: 496 bp for the *atpB* gene, 507 bp for the *EF-2* gene, 526 bp for the *glnA* gene, 514 bp for the *ppsA* gene, and 522 bp for the *rpoB'* gene (Table S3). Multitaxon alignments for the *EF-2* and *rpoB'* loci did not contain gaps, whereas several gaps were present within the *atpB, glnA*, and *ppsA* gene alignments. None of the positions in the alignments were omitted for the analysis.

### Phylogenetic tree reconstructions

Phylogenies were calculated for the 16S rRNA gene as well as for the individual housekeeping gene alignments and for the five concatenated loci. Optimal models of evolution were estimated from the nucleotide data using jModelTest version 2.1 (Darriba et al., 2012) considering 11 nucleotide substitution schemes, including models with equal/unequal base frequencies, with/without a proportion of invariable sites and with/without four rate variation among sites, and selecting the best model according to the Akaike (AIC) criterion (Akaike, 1974). The models proposed for the nucleotide data included TVM, TIM1, TIM2 (Posada, 2003), and GTR (Tavare, 1986). These models all consider unequal base frequencies, but vary in the number of transition and transversion rates deemed necessary to model evolution. All of the models proposed included the gamma shape parameter and considered invariable sites (Table S4).

The subsequent sequence analyses were performed using the PAUP^*^ version 4.0b10 phylogenetic software (Swofford, 2003) for the neighbor-joining (NJ) (Saitou and Nei, 1987) and maximum-parsimony (MP) (Kluge and Farris, 1969) methods and PhyML (Guindon and Gascuel, 2003) for the maximum-likelihood (ML) (Felsenstein, 1981) method. Support for NJ, MP, and ML phylogenies was determined through bootstrap analysis with 1,000 replications. Only bootstrap values equal or greater than 70% are shown on the trees. Topology congruence tests among individual and concatenated gene phylogenies were performed using Concaterpillar v. 1.8a software (Leigh et al., 2008) setting the *P*-value cutoff to 0.05.

A supertree, considering all previously obtained individual trees, was constructed on the basis of the five individual gene phylogenies by means of Matrix Representation using Parsimony (MRP) method (Loomis and Smith, 1990; Baum, 1992; Ragan, 1992) as implemented in Clann 3.2.3 (Creevey and McInerney, 2005) setting parameters by default.

### Lipid profile

Total lipids were extracted with chloroform/methanol using the extraction method described by Bligh and Dyer (1959), as modified for extreme halophiles (Corcelli et al., 2000). The extracts were carefully dried in a SpeedVac Thermo Savan SPD111V. The stock was prepared dissolving the dried extracts in chloroform to a final concentration 10 mg·ml-1. From this stock, 10 μl equivalent to 100 μg of total lipid extract were applied and analyzed by one dimensional High-Performance Thin Layer Chromatography (HPTLC) on Merck silica gel plates with solvent system A (chloroform: methanol: 90% acetic acid, 65: 4: 35, v/v) (Angelini et al., 2012). Staining of the lipids present in the HPTLC bands was carried out by spraying the plates with two different stains followed by brief heating at 160°C: (a) sulfuric acid 5% (v/v), a universal developer for visualizing all lipids; (b) molybdenum blue, specific for phospholipids.

### DNA–DNA hybridization, ANI calculation, and correlation studies

The strains used for DDH experiments included those isolated from the lake Aran-Bidgol and from the solar salterns in Namibia and Spain belonging to the MLSA defined groups 1, 2, and 3, and the species of the genus *Halorubrum* with validly published names that shared equal to or more than 97% 16S rRNA gene sequence similarity. Additionally, the type species of the genus *Halorubrum, Hrr. saccharovorum* DSM 1137^T^ was also included in the study as a reference. For DNA-DNA hybridizations strain Fb21 from group 1, strain Ib24 from group 2, and strain Cb34 from group 3 were randomly selected as representatives of each group and were used as reference for these studies.

DDH studies were conducted according to the competition procedure of the membrane filter method (Johnson, 1994), as previously reported for haloarchaea studies (Pesenti et al., 2008; Mancinelli et al., 2009; Corral et al., 2015). The hybridization temperature was 61.4°C, which was within the limit of validity for the filter method (De Ley and Tijtgat, 1970) and the percentage of hybridization was calculated according to Johnson (1994). The experiments were performed in triplicate. The interpretation is according to Wayne et al. (1987) where it has been established that strains belonging to the same species have values of DNA-DNA hybridization at or above 70% and Δ*Tm* equal or less than 5°C. Two strains having high values of DNA-DNA hybridization are phylogenetically related.

The *in silico* determined average nucleotide identity (ANI) between two genomes has been widely accepted by taxonomist as the substitute for DDH species delineation, with a cutoff value of 95–96% (Goris et al., 2007; Richter and Rosselló-Móra, 2009; Rosselló-Móra and Amann, 2015). ANI similarity index between pairs of genomes was calculated using BLAST (ANI_b_) as implemented in JSpecies software (Konstantinidis and Tiedje, 2005; Richter and Rosselló-Móra, 2009). The genome sequences of *Halorubrum* strains used for ANI_b_ calculation were retrieved from GenBank/EMBL/DDBJ databases or obtained in this study (Table S1).

DNA–DNA hybridization data obtained in this study were compared to the distance matrix data for the 16S rRNA gene, the distance matrix for each gene individually and the distance matrix corresponding to the 5-gene concatenated sequences, as well as to the ANI_b_ values. Correlation between values was calculated using Pearson's product–moment correlation coefficient.

## Results and discussion

### 16S rRNA gene sequence analyses

**Figure 1:**
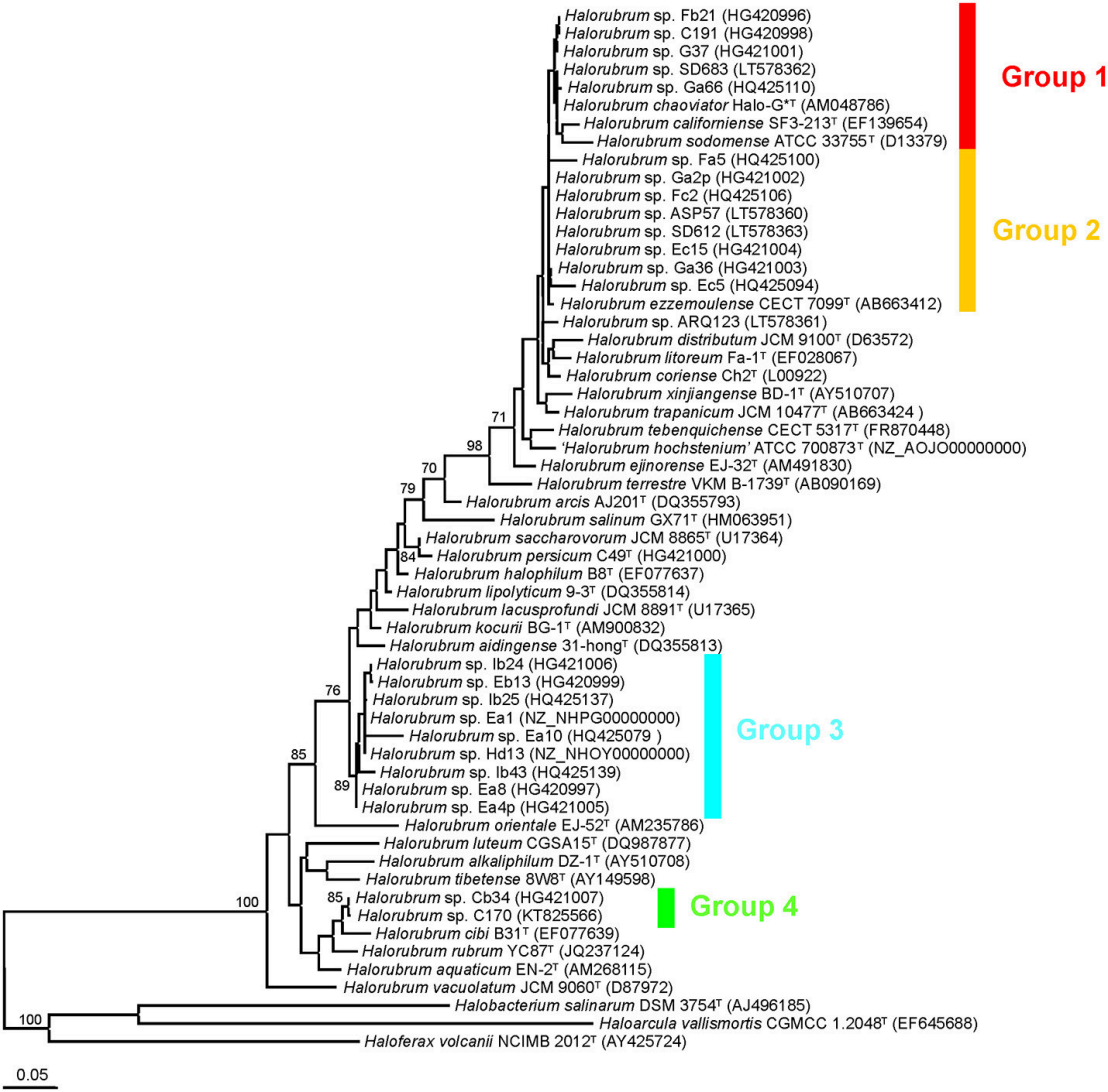
Maximum-likelihood tree based on 16S rRNA gene sequence showing the phylogenetic relationship between members of the genus *Halorubrum* and strains isolated in this study. The accession numbers of the sequences used are shown in parentheses after the strain designation. Bootstrap values >70% are indicated. The species *Haloarcula vallismortis, Haloferax volcanii*, and *Halobacterium salinarum* were used as outgroups. The scale bar represents 0.05 substitutions per nucleotide position. Different phylogroups have been marked with different colors.

Using samples obtained from the hypersaline lake Aran-Bidgol (Iran) and solar salterns in Namibia and Spain we were able to isolate 21, two, and two strains respectively (Table S1) that were affiliated with the genus *Halorubrum* according to their 16S rRNA and housekeeping gene sequences. In comparison with other *Halorubrum* type strains, the 16S rRNA gene phylogenetic trees showed that they formed four different phylogenetic clusters: group 1, comprising strains Fb21, C191, G37, SD683, and Ga66, and the species *Halorubrum chaoviator* Halo-G^*T^, *Halorubrum californiense* SF3-213^T^, and *Halorubrum sodomense* ATCC 33755^T^; group 2, including the Aran-Bidgol strains Fa5, Ga2p, Fc2, ASP57, SD612, Ec15, Ga36, and Ec5, as well the species *Halorubrum ezzemoulense* CECT 7099^T^; group 3, containing the Aran-Bidgol strains Ib24, Eb13, Ib25, Ea1, Ea10, Hd13, Ib43, Ea8, and Ea4p; and group 4, clustering the Aran-Bidgol strains Cb34 and C170. Strain ARQ123 fell in the boundaries of group 2, but did not belong to any of the above-identified phylogroups. Only groups 3 and 4 showed a strong bootstrap support (Figure 1). The 16S rRNA gene sequence similarities within each group were 100–99.7% for group 1, 100–98.0% for group 2, 100–98.3% for group 3, and 99.9% for group 4. Groups 1 and 2 were very closely related, sharing between 100–98.8% sequence similarities. The use of EzBioCloud database (Yoon et al., 2017) confirmed that for each group the most closely related taxa with validly published species names were: *Halorubrum chaoviator* DSM 19316^T^ for group 1 and group 2 (100% 16S rRNA gene sequence similarity in both cases); *Halorubrum kocurii* JCM 14978^T^ for group 3 (98.8%); and *Halorubrum cibi* B31^T^ for group 4 (98.9%). Despite the proximity of strain ARQ123 to group 2 observed in Figure 1, the EzBioCloud server indicated that the closest relative to strain ARQ123 was also the species *Halorubrum chaoviator* DSM 19316^T^, sharing 99.3% 16S rRNA gene sequence similarity. Applying the typically used 97% 16S rRNA gene species cutoff concept (Stackebrandt et al., 2002), the sequence data would suggest that all new isolates in this study should be considered as strains of previously described species that would not merit further analysis for the description of those groups as new species within the *Halobacteria*. However, 16S rRNA gene sequence conservation and transfer frequency conceals diversity in the class *Halobacteria* (Papke et al., 2004, 2007, 2011; Papke, 2009), suggesting the possibility of cryptic species in these *Halorubrum* strains. Therefore, we proceeded to investigate these *Halorubrum* strains with MLSA.

### MLSA analyses

For each locus phylogenetic trees with 1,000 bootstrap pseudoreplicates were constructed based on ML, MP and NJ methods, and according to the best evolutionary model calculated using jModelTest program (Table S4). Although all five alignments possessed a similar length (between 496 and 526 bp), *ppsA* had the highest proportion of parsimony-informative sites (39%), followed by *EF-2* (30%), *glnA* (27%), *rpoB'* (25%) and *atpB* (21%) genes (Table S3). The average pairwise sequence similarity values across the genus *Halorubrum* for *atpB, EF-2, glnA, ppsA*, and *rpoB'* were 95.0, 92.5, 95.5, 89.9, and 94.1%, respectively, suggesting that *ppsA* is the most resolving phylogenetic marker. Concatenation of the five loci produced an alignment of 2,565 bp, containing 28% parsimony-informative sites (Table S3), with an average pairwise sequence similarity of 93.7%.

**Figure 2:**
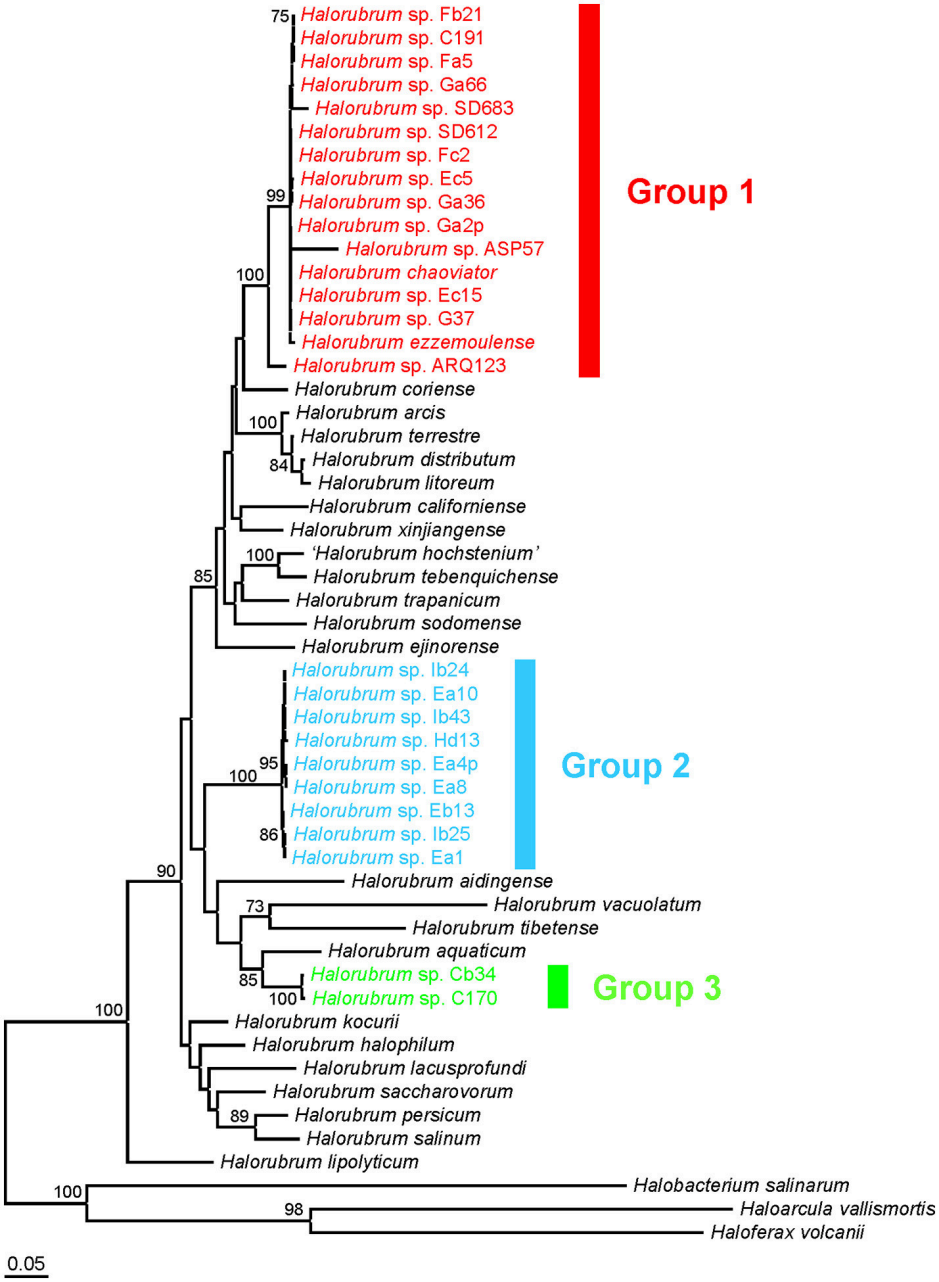
Maximum-likelihood tree based on the five-gene concatenated sequence showing the phylogenetic relationship between members of the genus *Halorubrum* and strains isolated in this study. The accession numbers of the sequences used are shown in Table S1. Bootstrap values >70% are indicated. The species *Haloarcula vallismortis, Haloferax volcanii*, and *Halobacterium salinarum* were used as outgroups. The scale bar represents 0.05 substitutions per nucleotide position. Different phylogroups have been marked with different colors.

**Figure 3:**
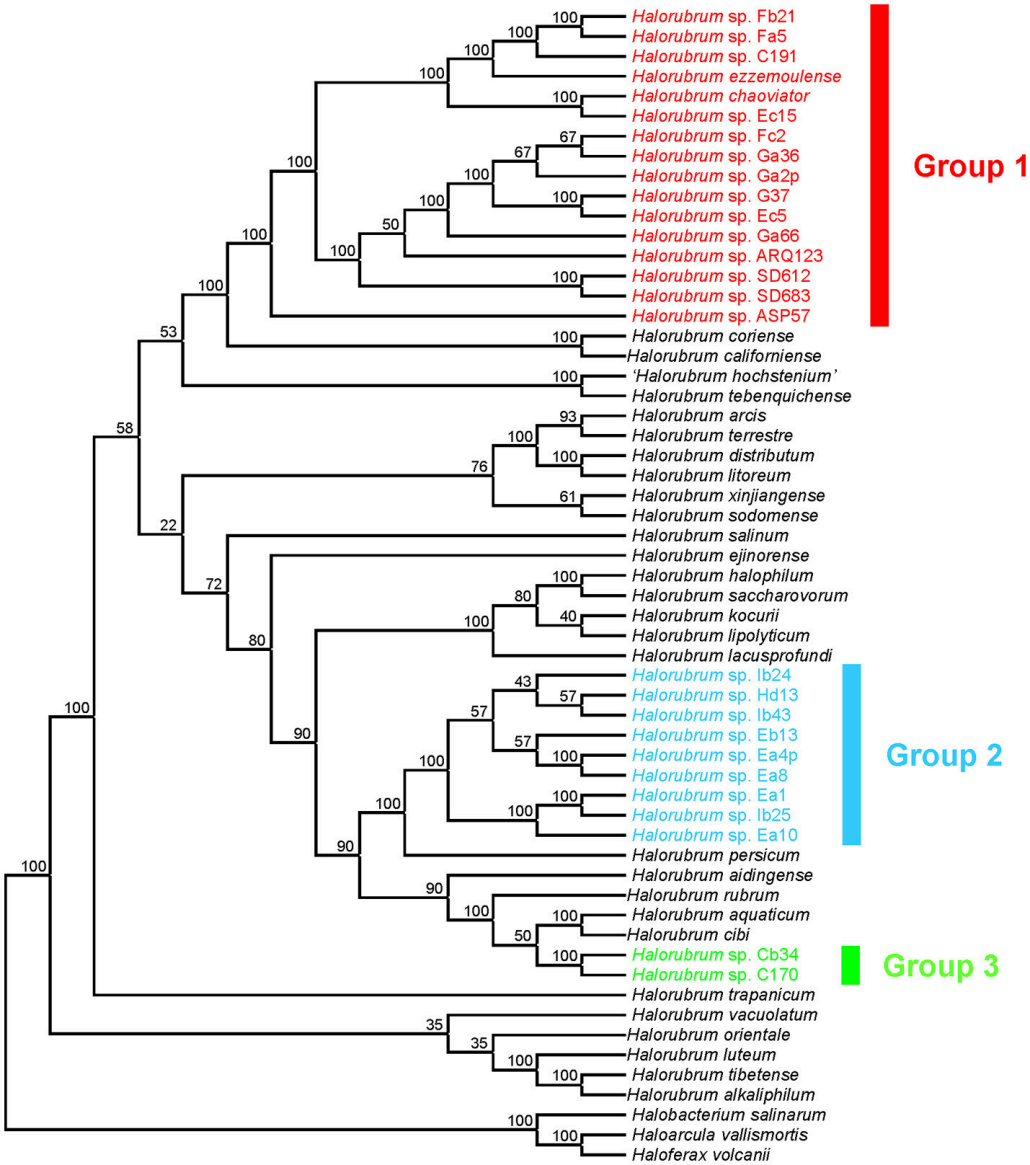
Consensus supertree constructed on the basis of the five individual gene phylogenies by means of Matrix Representation using Parsimony (MRP) method showing the phylogenetic relationship between members of the genus *Halorubrum* and strains isolated in this study. The numbers represent the proportion of universally distributed input supertrees that contained that particular branch in the consensus supertree. The species *Haloarcula vallismortis, Haloferax volcanii*, and *Halobacterium salinarum* were used as outgroups. Different phylogroups have been marked with different colors.

Phylogenetic trees constructed from individual protein coding genes (Figure S1), concatenated genes (Figure 2), and the supertree analysis (Figure 3) produced different overall topologies in comparison to each other, but they all support the inclusion of the same strains for groups 1, 2, and 3, with only two exceptions: in group 1, the *EF-2* and *rpoB'* phylogenies placed strains ARQ123 and ASP57, respectively, far from the other strains of group 1, which can be explained by gene transfer events. Groups 2 and 3 were consistently composed regardless of the analysis. The biggest difference between the protein coding and the 16S rRNA gene-based phylogenetic analyses was groups 1 and 2 in the 16S rRNA gene tree were collapsed into group 1 in the protein coding gene-based trees. *Halorubrum chaoviator* and *Halorubrum ezzemoulense* from groups 1 and 2 respectively, in the 16S rRNA gene tree, collapsed into a single large cluster called group 1 in the MLSA tree. *Halorubrum californiense* and *Halorubrum sodomense*, which fell into group 1 the rRNA gene tree were excluded from any of the new MLSA defined groups. Strain ARQ123, which was not part of groups 1 or 2 in the 16S rRNA gene tree found a home in group 1 in the concatenated and supertree analyses (Figures 2, 3).

Five-gene concatenated sequence similarities ranged between 100–95.8% for strains of group 1 (this range decreased to 100–98.8% when we excluded strains ARQ123 and ASP57 which have highly divergent *EF-2* and *rpoB'* genes respectively), 100–99.6% for group 2, and 99.7% for group 3 (Table 1). The most closely related type strains to group 1 were *Hrr. chaoviator* Halo-G^*T^/DSM 19316^T^ and *Hrr. ezzemoulense* DSM 17463^T^ (99.8% sequence similarity for both of them), to group 2 it was *Hrr. salinum* JCM 17093^T^ (94.4%), and to group 3 it was *Hrr. aquaticum* JCM 14031^T^/CGMCC 1.6377^T^ (95.0%) (Table 1); however, phylogenetic analyses indicated that the species *Hrr. salinum* JCM 17093^T^ is quite distantly related to group 2 strains in contrast to other *Halorubrum* species (Figures 2, 3). In comparison to the 16S rRNA gene sequence analysis, the MLSA approach provided clearer distinction between strains isolated in this study and previously described type strains, suggesting that groups 2 and 3 might each constitute new species. Because the MLSA analysis formed a monophyletic cluster inclusive of our group 1 new strains with two previously characterized and validly named species, *Hrr. chaoviator* Halo-G^*T^/DSM 19316^T^ and *Hrr. ezzemoulense* DSM 17463^T^, they could all comprise a single widely distributed species. The three strains used initially to characterize *Hrr. chaoviator* were isolated from Mexico, Australia and Greece (Mancinelli et al., 2009), and *Hrr. ezzemoulense* was cultivated from Algeria (Kharroub et al., 2006), which supports that conjecture.

**Table 1:** Similarity values for strains within groups 1, 2, and 3 based on the concatenated and individual housekeeping gene sequence and their most closely related taxa with validly published names.

**Strains**		**Similarity values within group (%)**	**Closest relative (% similarity)**
Concatenated	Group 1	100–95.8	*Hrr. chaoviator/Hrr. ezzemoulense* (99.8)
	Group 2	100–99.6	*Hrr. salinum* (94.4)
	Group 3	99.7	*Hrr. aquaticum* (95.0)
*atpB*	Group 1	100–94.5	*Hrr. chaoviator/Hrr. ezzemoulense* (100)
	Group 2	100–99.6	*Hrr. persicum* (97.1)
	Group 3	99.4	*Hrr. salinum* (94.9)
*EF-2*	Group 1	100–94.7	*Hrr. chaoviator*/*Hrr. ezzemoulense* (100)
	Group 2	100–99.0	*Hrr. kocurii*/*Hrr. lipolyticum* (94.9)
	Group 3	99.4	*Hrr. aquaticum* (95.7)
*glnA*	Group 1	100–99.2	*Hrr. chaoviator* (100)
	Group 2	100–99.4	*Hrr. distributum* (96.7)
	Group 3	100	*Hrr. aquaticum* (96.7)
*ppsA*	Group 1	100–99.2	*Hrr. ezzemoulense* (99.8)
	Group 2	100–99.4	*Hrr. chaoviator* (89.7)
	Group 3	99.8	*Hrr. aquaticum* (91.4)
*rpoB'*	Group 1	100–90.8	*Hrr. chaoviator*/*Hrr. ezzemoulense* (100)
	Group 2	100–99.8	*Hrr. xinjiangense* (96.2)
	Group 3	100	*Hrr. aquaticum*/*Hrr. rubrum* (96.7)

Although the concatenated (Figure 2) and the individual gene (Figure S1) phylogenies consistently clustered strains from this study into three groups, it appeared that their relationships with respect to the other *Halorubrum* strains (type and reference strains) might be different. Therefore, we compared the topology of each phylogenetic tree using the freely available program Concaterpillar (Leigh et al., 2008), and showed that there was substantial disagreement between trees (Table 2), which is a common observation caused by HGT (Martens et al., 2008; Pascual et al., 2010; Papke et al., 2011; de la Haba et al., 2012). In our phylogeny of *Halorubrum* isolates, the bootstrap analysis found support primarily for groups 1, 2, 3, and a few other shallow nodes, but very little support was found for many of the deeper nodes indicating an unknown relationship between species of *Halorubrum*. This poor resolution of relationships could be caused by many processes including rampant homoplasy and saturation of homologous sites, however it is well demonstrated that *Halorubrum* specifically (Papke et al., 2004, 2007) and all haloarchaea in general (Sharma et al., 2007; Papke et al., 2011; Nelson-Sathi et al., 2012; DeMaere et al., 2013) undergo tremendous amounts of homologous recombination and the more closely related two cells are the more frequent the transfer is between them (Naor et al., 2012; Williams et al., 2012). Therefore we suggest that the differences in tree topology reflect gene transfer rather than other evolutionary processes.

**Table 2:** Phylogenetic topology congruence analysis.

**Dataset**	**Concatenated**	***atpB***	***EF-2***	***glnA***	***ppsA***	***rpoB'***
Concatenated	1					
*atpB*	0.124	1				
*EF-2*	**0.016**	**0.001**	0.990			
*glnA*	**0.026**	**0.006**	**0.000**	1		
*ppsA*	0.455	**0.022**	**0.000**	**0.000**	0.999	
*rpoB'*	0.543	**0.015**	**0.000**	**0.016**	0.247	0.834

*Tree comparisons with a P-value < 0.05 are incongruent and bold highlighted*.

Although concatenated sequence alignments have proven to be a very useful method of reconstructing orthologous gene phylogenies, there are limitations to this approach, e.g., assumes that the same process of evolution has been acting on all the genes in the same manner. Therefore, we combined the phylogenetic relationships from the individual protein coding gene trees into an overall consensus supertree (Sanderson et al., 1998) and obtained a single phylogeny (Figure 3) by means of the MRP method. This strategy allowed us to analyse the same data through a very different set of assumptions and algorithms and yet the same groups were reconstructed, including that *Hrr. chaoviator* Halo-G^*T^/DSM 19316^T^ and *Hrr. ezzemoulense* DSM 17463^T^ clustered together with MLSA defined group 1 (Figure 3). Therefore, we have added evidence and confidence that each group is real, and that groups 2 and 3 may constitute new *Halorubrum* species. Interestingly, the supertree analysis distinguished subclusters that the MLSA tree did not. Consensus supertree group 1 strains forms two distinct clades each with 100% branch support. Within each, the Aran-Bidgol group 1 strains, with the lone exception of Ec15, are more closely related to each other than those from other locations, with 100% branch support.

### Lipid profiles

In the domain Archaea, polar lipid content was demonstrated to differentiate among taxa at the genus level, and sometimes at the species level (Torreblanca et al., 1986; Kates, 1993; Oren et al., 2009). In this study, we analyzed the lipid profile for strains isolated from the lake Aran-Bidgol and from the solar salterns in Namibia and Spain and their closest relatives and reference strains (*Halobacterium salinarum, Hrr. saccharovorum, Hrr. chaoviator, Hrr. ezzemoulense, Hrr. tibetense, Hrr. kocurii, Hrr. cibi* and *Natronococcus amylolyticus*) in order to further study, and possibly corroborate the obtained MLSA results. Since the lipid pattern may vary according to the culture conditions, a rigorous standardization of those conditions as well as of the starting quantity was applied to our analyses (see Methods).

**Figure 4:**
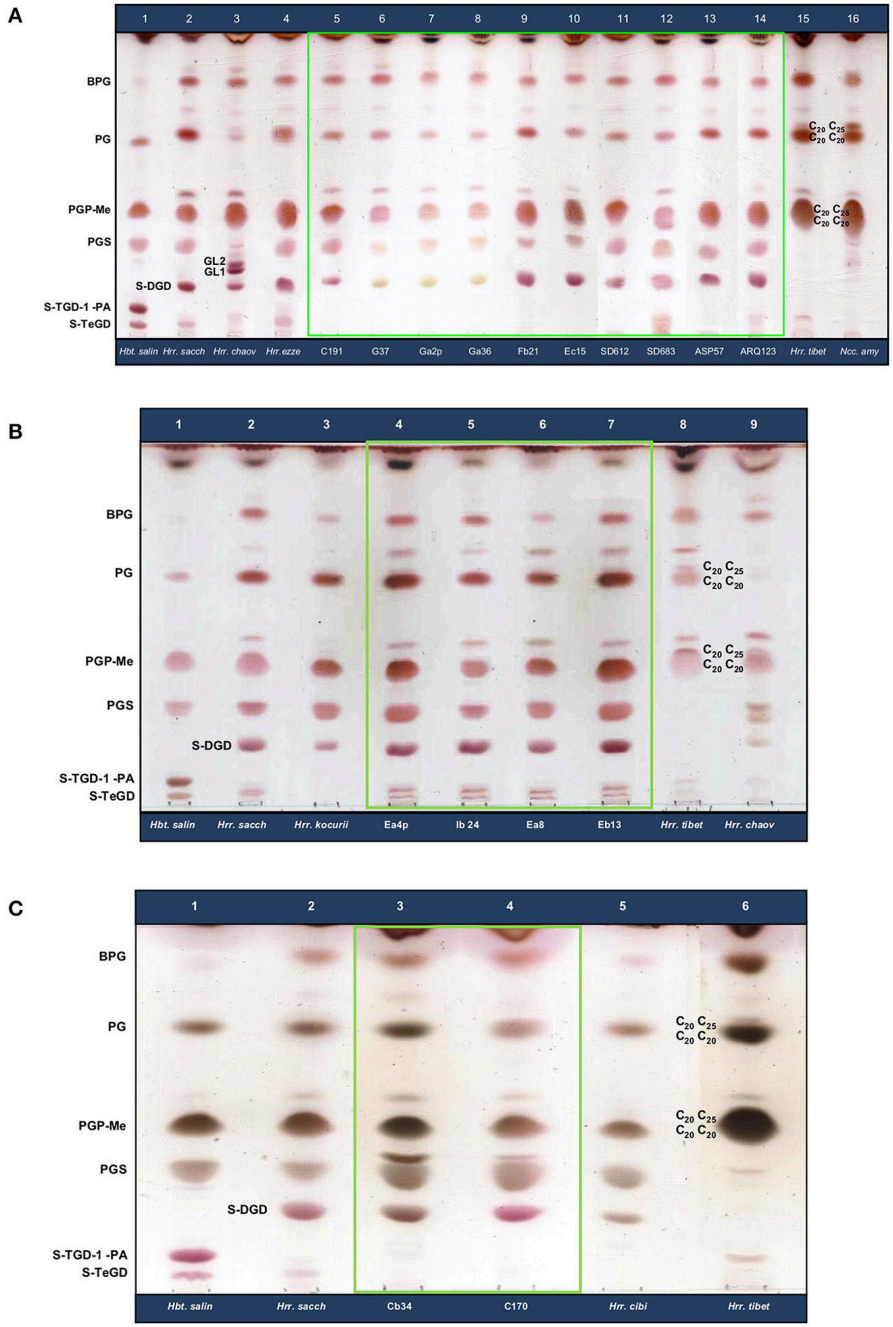
HPTLC stained with 5% (v/v) sulfuric acid showing the polar lipid profiles for *Halorubrum* strains belonging to group 1 and related taxa **(A)**, to group 2 and related taxa **(B)**, and to group 3 and related taxa **(C)**. *Hbt. salin, Halobacterium salinarum* DSM 3754^T^; *Hrr. sacch, Halorubrum saccharovorum* DSM 1137^T^; *Hrr. chaov, Halorubrum chaoviator* Halo-G^*^^T^; *Hrr. ezze, Halorubrum ezzemoulense* DSM 17463^T^; *Hrr. tibet, Halorubrum tibetense* JCM 11889^T^; *Ncc. amy, Natronococcus amylolyticus* DSM 10524^T^; *Hrr. kocurii, Halorubrum kocurii* CECT 7322^T^; *Hrr. cibi, Halorubrum cibi* JCM 15757^T^. BPG, biphosphatidylglycerol; PG, phosphatidylglycerol; PGP-Me, phosphatidylglycerolphosphate methyl ester; PGS, phosphatidylglycerolsulfate; GL, unidentified glycolipid; S-DGD, sulfated mannosyl glucosyl diether; S-TGD-1-PA, sulfated galactosyl mannosy glucosyl dietherphosphatidic acid; S-TeGD, sulfated tetraglycosyl diether.

HPTLC results showed that strains from the lake Aran-Bidgol and from the solar salterns in Namibia and Spain belong to the genus *Halorubrum*, showing the characteristic lipids of this genus growing optimally at neutral pH values (McGenity and Grant, 2001; Oren et al., 2009). All strains belonging to group 1 possess C_20_C_20_ and C_20_C_25_ derivates of phosphatidylglycerolphosphate methyl esther (PGP-Me), phosphatidylglycerolsulphate (PGS), C_20_C_20_ derivates of phosphatidylglycerol (PG) and biphosphatidylglycerol (BPG) as the main polar lipids. A sulphated glycolipid similar to sulphated mannosyl glycosyl diether (S-DGD) was also detected in group 1 strains and a minor co-migratory band with BPG was also present in some of these strains (Figure 4A and Figure S2A). Two haloalkaliphilic species, *Hrr. tibetense* JCM 11889^T^ and *Natronococcus amylolyticus* DSM 10524^T^ were included in this study with the aim of comparing the presence of the double chain length C_20_C_20_ and C_20_C_25_ derivates of PG not typically found in neutrophilic species of *Halorubrum*. The species *Hrr. chaoviator* Halo-G^*T^ and *Hrr. ezzemoulense* DSM 17463^T^, showed lipid profiles similar to our isolated strains of group 1, but were not identical. The type strain *Hrr. chaoviator* Halo-G^*T^ presented GL1 and GL2, which is absent in all the other strains of group 1 (Figure 4A and Figure S2A). Additionally, all group 1 Aran-Bidgol strains lacked a minor spot at the bottom of the HPTLC plate that was present in the Namibian and Spanish strains as well as in the species *Hrr. chaoviator* Halo-G^*T^ and *Hrr. ezzemoulense* DSM 17463^T^. These differences in lipid profiles demonstrate well the phenotypic and likely genotypic plasticity within group 1. Further, given that the group 1 Aran-Bidgol strains profiles are more similar to each other than they are to the rest of group 1, we propose that the Aran-Bidgol group 1 strains are evolving in concert, and separately from the other group 1 strains.

Lipid analysis of Aran-Bidgol strains that form group 2, and its closest relative *Hrr. kocurii* CECT 7322^T^ also demonstrated a clearly differentiated lipid pattern. All Aran-Bidgol group 2 strains had the same profile: a minor phosphoglycolipid below the S-DGD spot, a minor phospholipid near the PGP-Me spot, and a minor glycolipid close to the PG spot, which are all absent in the *Hrr. kocurii* CECT 7322^T^ profile (Figure 4B and Figure S2B). Therefore, the MLSA differences observed between group 2 and its closest known validly named species *Hrr. kocurii* are further corroborated by the lipid analysis providing additional evidence that these strains likely constitute a new *Halorubrum* lineage. Nevertheless, more work needs to be done to corroborate the validity of this statement.

Both strains included in group 3 showed the same lipid pattern, which was different in comparison to their most closely related species, *Hrr. cibi* JCM 15757^T^. The main difference is the presence of minor phospholipids as co-migratory bands above the PGP-Me and PGS spots, respectively, in contrast with *Hrr. cibi* JCM 15757^T^, where these minor lipids cannot be observed (Figure 4C and Figure S2C). Again, the lipid profile agrees with the MLSA data and supports the placement of Aran-Bidgol strains forming group 3 as a different lineage than that of *Hrr. cibi* JCM 15757^T^, although a more extensive analysis including the polar lipids of all the species in the genus *Halorubrum* would be required prior to become widespread the use of the lipid profile as a discriminating approach for species delineation within this genus.

### DNA–DNA hybridization and ANI

Results of DDH indicated that group 1 Aran-Bidgol strains Fb21, C191, Ec15, G37, Ga2p, and Ga36 are homogeneous with respect to their reassociation values: they are all equal to or higher than 70% in comparison to the group 1 reference strain Fb21. This result indicates they belong to the same taxonomic species (Table 3). The ANI values between those strains were all 96.6% or higher (Table 3), which is above the 95–96% cutoff limit for species delineation (Goris et al., 2007; Richter and Rosselló-Móra, 2009; Rosselló-Móra and Amann, 2015). Therefore, the DDH and ANI data are in agreement that these Aran-Bidgol strains should be classified as belonging to the same species. On the other hand, the DDH analysis does not support Aran-Bidgol group 1 strains as belonging to the previously described and closely related *Hrr. ezzemoulense* (22%) and *Hrr. chaoviator* (54%), which could not be distinguished as different species based on MLSA or ANI (Table 3). In order to confirm these results, the strain *Hrr. ezzemoulense* DSM 17463^T^ was used as reference for DDH experiments within group 1, showing 91 and 79% reassociation values with the Spanish strains ASP57 and ARQ123, 89 and 86% with the Namibian strains SD683 and SD612, and 79% with the named species *Hrr. chaoviator* HaloG^*T^, but 26, 10 and 1% with the Aran-Bidgol strains Ga36, Ga2p and G37, respectively. The value differences between ANI and DDH likely reflect accessory genome content evolution, which is known to change quickly between closely related *Halorubrum* strains (Ram Mohan et al., 2014) and others (Welch et al., 2002; Thompson et al., 2005). Although MLSA and ANI group Aran-Bidgol, Spanish and Namibian strains, as well as *Hrr. chaoviator* Halo-G^*T^/DSM 19316^T^ and *Hrr. ezzemoulense* DSM 17463^T^ into a single species, it is clear that the DDH results are in agreement with the polar lipid profiles that indicated Aran-Bidgol group 1 strains are homogeneous, but clearly different from *Hrr. chaoviator* Halo-G^*T^/DSM 19316^T^ and *Hrr. ezzemoulense* DSM 17463^T^ strains.

**Table 3:** DNA–DNA hybridization data (%) among representative strains of groups 1 (Fb21), 2 (Ib24), and 3 (Cb34) and its closest relatives.

**GROUP 1**				
**Competitor DNA**	**DDH with strain Fb21**	**16S rRNA gene similarity (%) with Fb21**	**Five concatenated gene similarity (%) with Fb21**	**ANI**_b_**-values (%) with Fb21**
*Halorubrum* sp. Fb21	100	100	100	100
*Halorubrum* sp. C191	71	100	100	98.3
*Halorubrum* sp. G37	99	99.9	99.8	97.0
*Halorubrum* sp. SD683	ND	99.9	98.9	97.2
*Halorubrum* sp. SD612	ND	99.8	99.7	97.3
*Halorubrum chaoviator* DSM 19316^T^	54	99.8	99.8	98.5
*Halorubrum* sp. Ec15	75	99.6	99.7	96.8
*Halorubrum* sp. Ga2p	99	99.6	99.8	96.6
*Halorubrum* sp. Ga36	98	99.6	99.7	96.7
*Halorubrum* sp. ARQ123	ND	99.1	98.0	ND
*Halorubrum* sp. ASP57	ND	99.1	97.5	ND
*Halorubrum ezzemoulense* DSM 17463^T^	22	99.1^a^	99.7	98.3
*Halorubrum californiense* CECT 7256^T^/SF3 213^T^/DSM 19288^T^	43	99.1	94.3	87.3
*Halorubrum trapanicum* NRC 34021^T^	3	99.0	94.4	87.2
*Halorubrum coriense* JCM 9275^T^/Ch2^T^/DSM 10284^T^	21	98.9	95.4	88.0
*Halorubrum sodomense* JCM 8880^T^/DSM 3755^T^/RD 26^T^	3	98.9	93.7	89.5
*Halorubrum xinjiangense* JCM 12388^T^/CGMCC 1.3527^T^	15	98.6	94.7	90.1
*Halorubrum litoreum* JCM 13561^T^	31	98.5	95.2	87.7
*Halorubrum tebenquichense* CECT 5317^T^/DSM 14210^T^	18	98.3	94.5	85.5
*Halorubrum distributum* JCM 10118T/JCM 9100^T^	49	98.2	95.2	87.8
*Halorubrum ejinorense* CECT 7194^T^/EJ-32^T^	38	98.1	93.5	ND
*Halorubrum terrestre* JCM 10247^T^/VKM B-1739^T^	9	97.2	95.1	87.6
*Halorubrum saccharovorum* DSM 1137^T^/JCM 8865^T^	7	95.7	92.9	81.9
**GROUP 2**				
**Competitor DNA**	**DDH with strain Ib24**	**16S rRNA gene similarity (%) with Ib24**	**Five concatenated gene similarity (%) with Ib24**	**ANI**_b_**-values (%) with Ib24**
*Halorubrum* sp. Ib24	100	100	100	100
*Halorubrum* sp. Eb13	76	99.9	99.8	98.7
*Halorubrum* sp. Ea8	83	99.5	99.8	98.3
*Halorubrum* sp. Ea4p	73	99.5	99.8	ND
*Halorubrum kocurii* CECT 7322^T^/JCM 14978^T^	37	98.3	93.5	85.5
*Halorubrum lipolyticum* JCM 13559^T^/9-3^T^/DSM 21995^T^	7	98.2	91.4	85.7
*Halorubrum aidingense* JCM 13560^T^/31-hong^T^	9	98.1	91.1	83.9
*Halorubrum halophilum* B8^T^	ND	97.8	93.2	84.1
*Halorubrum lacusprofundi* JCM 8891^T^/ATCC 49239^T^	14	97.6	91.6	84.9
*Halorubrum saccharovorum* DSM 1137^T^/JCM 8865^T^	1	97.5	93.4	85.5
**GROUP 3**				
**Competitor DNA**	**DDH with strain Cb34**	**16S rRNA gene similarity (%) with Cb34**	**Five concatenated gene similarity (%) with Cb34**	**ANI**_b_**-values (%) with Cb34**
*Halorubrum* sp. Cb34	100	100	100	100
*Halorubrum* sp. C170	100	99.9	99.7	ND
*Halorubrum cibi* JCM 15757^T^/B31^T^	42	98.9	ND	ND
*Halorubrum aquaticum* EN-2^T^/CGMCC 1.6377^T^	59	98.5	95.0	87.8
*Halorubrum alkaliphilum* JCM 12358^T^/DZ-1^T^	51	97.3	ND	ND
*Halorubrum tibetense* JCM 11889^T^/8W8^T^	56	97.3	91.3	ND
*Halorubrum kocurii* CECT 7322^T^/JCM 14978^T^	57	97.2	93.6	81.5
*Halorubrum lipolyticum* JCM 13559^T^/9-3^T^/DSM 21995^T^	50	97.1	90.4	81.8

*Halorubrum species for DDH experiments were selected according to their 16S rRNA gene sequence similarity (calculated using EzBioCloud) respect to the representative strain of groups 1, 2, and 3. Five concatenated gene sequence pairwise comparison values and ANI_b_-values are also shown. ND, not determined*.

a *16S rRNA gene sequence similarity calculated using BLAST and the sequence AB663412 for Hrr. ezzemoulense CECT 7099^T^, since the sequence available on EzBioCloud database (accession number DQ118426) was a low quality one*.

Similar DDH results were obtained in analyzing the other strains from Aran-Bidgol: strains within the phylogenetic clusters 2 and 3 were homogeneous with respect to each other, having reassociation values higher than 70% and being unmistakably separated from closely related validly named species with values below the 70% threshold. ANI values were in agreement with the MLSA and DDH analysis for groups 2 and 3 being differentiated from all known validly named species, indicating these likely constitute new species.

Another widely accepted alternative to experimental DDH and ANI for prokaryotic species circumscription is the so-called *in silico* DDH (Auch et al., 2010). However, since ANI_b_ values > 75% show similar results/interpretations as *in silico* DDH (Li et al., 2015), it was not taken into consideration in the present study.

### Correlation studies

**Figure 5:**
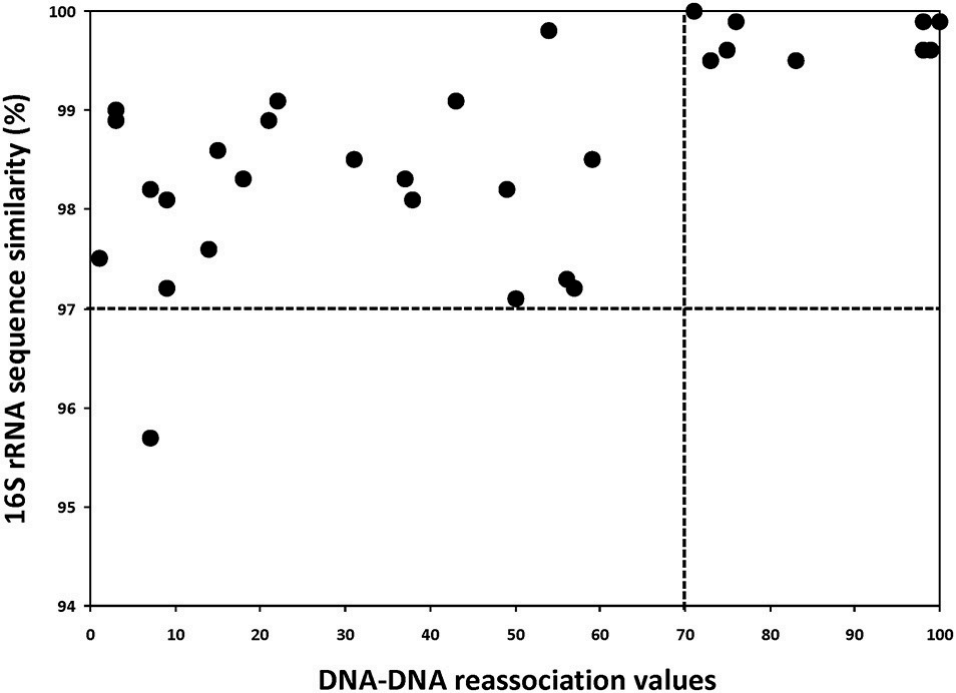
Graph of the 16S rRNA gene sequence similarity vs. DDH relatedness values for the genus *Halorubrum*. The vertical dashed line indicates the 70% DDH threshold, while the horizontal dashed line indicates 97% 16S rRNA gene sequence similarity.

**Figure 6:**
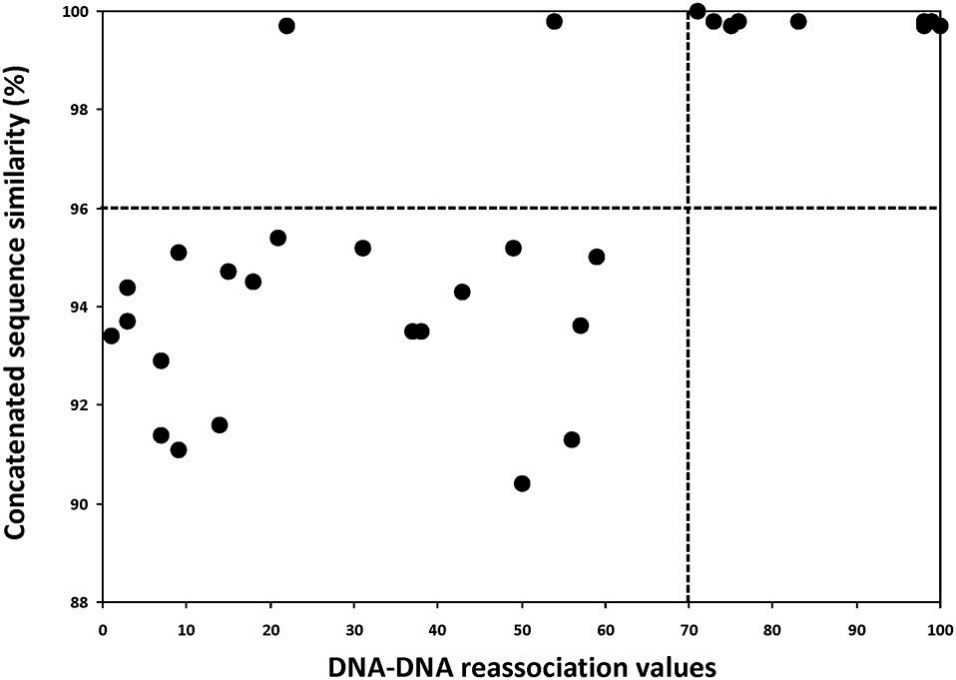
Graph of the five concatenated gene sequence similarity vs. DDH relatedness values for the genus *Halorubrum*. The vertical dashed line indicates the 70% DDH threshold, while the horizontal dashed line indicates the proposed genetic similarity threshold for distinguishing genomic species. The two points above the 96% limit and below 70% DDH are pairwise comparisons of *Hrr. chaoviator* Halo-G^*^^T^/DSM 19316^T^ vs. *Halorubrum* sp. Fb21 and *Hrr. ezzemoulense* DSM 17463^T^ vs. *Halorubrum* sp. Fb21.

To gain a broader understanding for the different molecular techniques applied in this study, we compared the DDH data against the 16S rRNA gene sequence similarities (Figure 5) and against MLSA data (Figure 6) and calculated the correlation values. As observed in Figure 5, the traditional threshold value of 97% for 16S rRNA gene sequences is not useful for delineating species. All but one *Halorubrum* species having less than 70% DDH reassociation values had greater than 97% 16S rRNA gene sequence similarity, the majority had greater than 98% similarity and four had 99% or more (Table 3). Of particular interest was that strains exhibiting nearly 100% DDH reassociation values did not have 100% 16S rRNA gene sequence similarity (suggesting evidence for 16S rRNA gene HGT) (Table 3). On the other hand, comparison of DDH values and MLSA gene concatenation showed that having less than 96% sequence similarity indicates strains are different species. A stricter cutoff of 99% might be feasible based on the clustering of points above 99% sequence similarity, and absence of data between 96 and 99% for MLSA defined species (Figure 6). However, of special consideration is that the threshold values only demarcates unambiguously that two strains belong to separate species, which is not equivalent to saying that strains with greater than 96% (or 99%) belong to the same species.

The Pearson's product-moment correlation coefficient was calculated between the DDH relatedness matrix and the corresponding similarity matrices of the 16S rRNA gene, the five individual genes, the concatenation of the five genes and the ANI values of the sequenced genomes. Each comparison correlated between 33 and 27 pairs of values. The coefficients obtained were 0.58 for the 16S rRNA gene, and 0.57 for *ppsA*, 0.63 for *atpB*, 0.64 for *rpoB'*, 0.73 for *glnA*, and 0.74 for *EF-2*. The corresponding coefficient for the five concatenated genes was 0.70 and for the ANI value it was 0.65. Therefore, an acceptable correlation between DDH and evolutionary distances is observed, similar to those observed for *Streptomyces griseus* (Rong and Huang, 2010), *Vibrio* (Pascual et al., 2010) and *Salinivibrio* (López-Hermoso et al., 2017). However, though the correlation is acceptable, it is not as high as in those studies. One reason could be the low DDH values obtained between Fb21 and *Hrr. chaoviator* DSM 19316^T^ and *Hrr. ezzemoulense* DSM 17463^T^. Reanalysis that excluded comparison of the two type strains with Fb21 demonstrated a rise in the correlation coefficient for all genes or genomes: 0.60 for 16S rRNA gene, 0.60 for *ppsA*, 0.69 for *atpB*, 0.70 for *rpoB'*, 0.80 for *glnA*, 0.83 for *EF-2*, and 0.77 and 0.73 for the concatenated sequences and the ANI values, respectively.

## Conclusions

In this study we used an MLSA approach to infer phylogenetic relationship among the species of the genus *Halorubrum* and we have applied this scheme to analyze many new isolates. The MLSA results were complemented with 16S rRNA gene sequencing, lipid profiling, DDH, and ANI analyses. While it is clear that all data presented are in agreement regarding groups 2 and 3 as belonging to new species, they were tentative for group 1. On the basis of MLSA and ANI results, it is demonstrated that all isolated strains of group 1 (from Aran-Bidgol lake and Namibian and Spanish solar salterns, and the previously named species *Hrr. chaoviator* Halo-G^*T^/DSM 19316^T^ and *Hrr. ezzemoulense* DSM 17463^T^) constitute a single species. A lack of clarity develops from the observed DDH values and lipid profiling which indicated that group 1 Aran-Bidgol strains are all more similar to each other, yet different from the other group 1 strains, and could belong to separate species if DDH and PLP analyses have more weight than sequence data. Historically and contemporaneously, DDH is the gold standard for species inclusion/exclusion, and polar lipids provide a diagnostic phenotype to separate them, indicating the MLSA defined and ANI validated group 1 is likely composed of more than one taxonomic species.

Uniqueness in taxonomic classification likely hinges on the consideration of evolutionary forces that homogenize diversity within populations, and that generate diversity between them. All of the Aran-Bidgol strains forming group 1 are genetically homogeneous according to their MLSA, ANI, DDH and lipids, while strains from the same MLSA cluster but cultivated from Mexico, Algeria, Namibia and Spain, demonstrate clear differences. This indicates that *Hrr. ezzemoulense, Hrr. chaviator* and strains from Namibia (SD612 and SD683) and Spain (ARQ123 and ASP57) are not undergoing the same homogenizing forces as the population from Aran-Bidgol. Based on previous analyses of gene flow in *Halorubrum* spp. and other haloarchaea (Papke et al., 2011; Naor et al., 2012; Nelson-Sathi et al., 2012; Williams et al., 2012; DeMaere et al., 2013), HGT is a strong candidate for the evolutionary forces homogenizing the Aran-Bidgol groups. Since *Hrr. ezzemoulense, Hrr. chaviator*, strains SD612 and SD683 and strains ARQ123 and ASP57 were each cultivated from different locations this may not be surprising, as hypersaline environments are patchily distributed microbial “Galapagos” islands, that the differences observed may reflect “slow” migration between sites relative to the rate of evolutionary change. Previous analyses of the Aran-Bidgol strains support our hypothesis: they are losing and adding variability through gene loss and HGT possibly at rates faster than the accumulation of substitutions at redundant codon positions in MLSA genes (Fullmer et al., 2014; Ram Mohan et al., 2014). Therefore, our data support the interpretation that there is an absence of a worldwide homogenizing force, and separated populations are able to acquire localized variation and diverge, which promotes speciation. Additional data supporting our hypothesis is that the three strains used to characterize *Hrr. chaoviator*, which were cultivated from three different geographic locations, also had different lipid profiles (Mancinelli et al., 2009). Our observations also indicate the capacity for rapid adaptation to a new location once arrived, as our lipids analysis shows high variability among cluster 1 strains that were cultivated from different locations. Limitations to haloarchaeal dispersal and invasion are further validated by recent analyses showing that different locations distributed around the globe, and even ones within the same country, have substantially different community compositions (Oh et al., 2010; Dillon et al., 2013; Zhaxybayeva et al., 2013; Fernández et al., 2014b). Our data are in agreement with those studies, indicating that limitations to dispersal and invasiveness may be primary drivers of haloarchaeal divergence and speciation.

There has long been a debate in the taxonomy and systematics fields over whether or not multiple strains should be required for classifying new species. It is worthwhile pointing out that our conclusions would be dampened immensely if we had not analyzed many closely related strains: the homogeneity observed in the Aran-Bidgol group 1 population would have gone undetected, or might have been considered a one-off result. Our population data showing that all the group 1 Aran-Bidgol strains were similar to each other and different from their closely related kin reinforced our conclusions regarding their differences and amplifying the importance of using multiple strains. Including evidence from natural populations to guide taxonomy appears to boost the robustness and accuracy in classification and should be considered as important as the methodological aspects to classification.

Our study establishes the usefulness of an MLSA approach for distinguishing between species within the genus *Halorubrum*. We were able to demonstrate a 4% MLSA sequence divergence cutoff correlates with the 70% DDH gold standard of prokaryotic taxonomy, thus reducing the necessity for performing DDH in future taxonomic studies: in the case of strains obtained from different geographic locations with high sequence similarity it appears DDH and other analyses would still provide useful taxonomic insight. The advantages of using an MLSA approach over DDH are enormous: sequence data can be stored for all downstream discoveries of new species; it is less prone to error; it does not require radioisotopes or other tedious methodologies; and it can provide useful evolutionary relationships. While 16S rRNA gene sequence analysis is storable and capable of phylogenetic reconstruction in the haloarchaea, we have shown conclusively that a follow up DDH analysis is nearly certainly needed to distinguish any new species. This does not appear to be the case for the MLSA. Further, several genera of the haloarchaea (e.g., *Halomicrobium, Haloarcula, Halosimplex, Halomicroarcula, Haloarchaeobius*) carry multiple highly divergent rRNA operons and are known to be extremely prone to PCR artifacts and thus erroneous identification and classification (Boucher et al., 2004; Zhang and Cui, 2014a,b). Therefore, in agreement with the recommendations of the ICSP-Subcommittee on the taxonomy of *Halobacteria* (Oren and Ventosa, 2013, 2016), we strongly encourage the use of our MLSA approach for descriptions of novel species within this family. For practical matters of classification, we propose the 4% MLSA nucleotide sequence dissimilarity threshold value for unequivocally distinguishing new genomic species for the haloarchaea, with the caveat that strains having less concatenated sequence divergence could also be different species and would need to be tested by DDH or genomic indexes for certainty.

## Author contributions

AV, RP, CS-P, and RH conceived and designed the study. PC, CI-D, CS-P, MA, AV, and RP designed and performed the acquisition of environmental isolates. RH, PC, CS-P, CI-D, and AM performed the microbial experiments. RH, PC, CS-P, CI-D, AM, AV, and RP analyzed and interpreted the data. RH, PC, CS-P, CI-D, AM, MA, AV, and RP discussed the paper. RH and PC drafted the paper. RH, PC, CS-P, CI-D, AM, MA, AV, and RP critically revised the manuscript. All authors read and approved the final manuscript.

### Conflict of interest statement

The authors declare that the research was conducted in the absence of any commercial or financial relationships that could be construed as a potential conflict of interest.
