# Supplementary material for: Genotypic and Lipid Analyses of Strains From the Archaeal Genus Halorubrum Reveal Insights Into Their Taxonomy, Divergence, and Population Structure
Source: Front Microbiol. 2018 Mar 29;9:512. doi: 10.3389/fmicb.2018.00512 (PMC5890160; doi:10.3389/fmicb.2018.00512)
Supplement: Supplementary file 1 [file Table1.doc]

***Supplementary Material***

**Genotypic and lipid analyses of strains from the archaeal genus *Halorubrum* reveal insights into their taxonomy, divergence and population structure**

**Rafael R. de la Haba^¶^, Paulina Corral^¶^, Cristina Sanchez-Porro, Carmen Infante-Domínguez, Andrea M. Makkay, Mohammad A. Amoozegar, Antonio Ventosa^*^, R. Thane Papke^*^**

**^*^ Correspondence:**

Dr. Antonio Ventosa

[ventosa@us.es](mailto:ventosa@us.es)

Dr. R. Thane Papke

[thane@uconn.edu](mailto:thane@uconn.edu)

**^¶^**The two first authors equally contributed to this work.

**Supplementary Figures**

**A**

**B**

**C**

**D**

**E**

**Supplementary Figure S1. Maximum-likelihood tree based on the *atpB* (A), *EF-2* (B), *glnA* (C), *ppsA* (D) and *rpoB’* (E) gene sequences showing the phylogenetic relationship between members of the genus *Halorubrum* and strains isolated in this study.** Bootstrap values >70 % are indicated. The species *Haloarcula vallismortis*, *Haloferax volcanii* and *Halobacterium salinarum* were used as outgroups. The scale bar represents 0.02, 0.05, 0.1, 0.1 and 0.05 substitutions per nucleotide position, respectively. Different phylogroups have been marked with different colours.

**A**

**B**

**C**

**Supplementary Figure S2. High-performance thin layer chromatography (HPTLC) stained with molibdenum blue showing the polar lipid profiles for *Halorubrum* strains belonging to group 1 and related taxa (A), to group 2 and related taxa (B) and to group 3 and related taxa (C).** *Hbt. salin*, *Halobacterium salinarum* DSM 3754^T^; *Hrr. sacch*, *Halorubrum saccharovorum* DSM 1137^T^; *Hrr. chaov*, *Halorubrum chaoviator* Halo-G*; *Hrr. tibet*, *Halorubrum tibetense* JCM 11889^T^; *Ncc. amy*, *Natronococcus amylolyticus* DSM 10524^T^; *Hrr. kocurri*, *Halorubrum kocurii* CECT 7322^T^; *Hrr. cibi*, *Halorubrum cibi* JCM 15757^T^. BPG, biphosphatidylglycerol; PG, phosphatidylglycerol; PGP-Me, phosphatidylglycerolphosphate methyl ester; PGS, phosphatidylglycerolsulfate; GL, unknown glycolipid; S-DGD, sulfated mannosyl glucosyl diether; S-TGD-1-PA, sulfated galactosyl mannosy glucosyl dietherphosphatidic acid; S-TeGD, sulfated tetraglycosyl diether.

**Supplementary Tables**

**Supplementary Table S1. Analyzed strains of the genus *Halorubrum* and other reference strains within the class *Halobacteria* used in this study showing the accession numbers of sequences deposited in GenBank/EMBL/DDBJ.** Sequences marked in bold are from this study. ND, not determined.

| **Strain** | ***atpB*** | ***EF-2*** | ***glnA*** | ***ppsA*** | ***rpoB’*** | **16S rRNA** | **Genome** |
| --- | --- | --- | --- | --- | --- | --- | --- |
| *Haloarcula vallismortis* ATCC 29715^T^/CGMCC 1.2048^T^ | HQ149351 | HQ149398 | **KM107962** | **KM107931** | NZ_AOLQ00000000 | EF645688 | NZ_AOLQ00000000 |
| *Halobacterium salinarum*  R1^T^/DSM 3754^T^ | AM774415 | AM774415 | AM774415 | AM774415 | AM774415 | AJ496185 | NC_010364 |
| *Haloferax volcanii* DS2^T^/NCIMB 2287^T^/NCIMB 2012^T^ | CP001956 | HQ149404 | CP001956 | CP001956 | HQ149487 | AY425724 | CP001956 |
| *Halorubrum aidingense* JCM 13560^T^/31-hong^T^ | HQ149374 | HQ149406 | **KM107963** | NZ_AOJI00000000 | AB477176 | DQ355813 | NZ_AOJI00000000 |
| *Halorubrum alkaliphilum* JCM 12358^T^/DZ-1^T^ | ND | ND | ND | ND | AB477177 | AY510708 | ND |
| *Halorubrum aquaticum* JCM 14031^T^/CGMCC 1.6377^T^ | PRJNA303461 | KM980074 | PRJNA303461 | PRJNA303461 | AB820296 | AM268115 | PRJNA303461 |
| *Halorubrum arcis*  JCM 13916^T^/AJ201^T^ | **KM105848** | **KM107920** | **KM107964** | **KM107932** | AB477178 | DQ355793 | NZ_AOJJ00000000 |
| *Halorubrum californiense* DSM 19288^T^/SF3-213^T^ | ND | NZ_AOJK00000000 | NZ_AOJK00000000 | NZ_AOJK00000000 | NZ_AOJK00000000 | EF139654 | NZ_AOJK00000000 |
| *Halorubrum chaoviator* Halo-G*^T^/DSM 19316^T^ | **KM105849** | **KM107921** | **KM107965** | **KM107933** | AB820293 | AM048786 | NZ_NDWV00000000 |
| *Halorubrum cibi* JCM 15757^T^/B31^T^ | ND | ND | ND | ND | AB820297 | EF077639 | ND |
| *Halorubrum coriense*  JCM 9275^T^/DSM 10284^T^/Ch2^T^ | HQ149357 | HQ149407 | **KM107966** | **KM107934** | HQ149490 | L00922 | NZ_AOJL00000000 |
| *Halorubrum distributum* JCM 9100^T^ | HQ149358 | HQ149408 | **KM107967** | **KM107935** | HQ149491 | D63572 | NZ_AOJM00000000 |
| *Halorubrum ejinorense* JCM 14265^T^/EJ-32^T^ | **KM105850** | **KM107922** | **KM107968** | **KM107936** | AB820295 | AM491830 | ND |
| *Halorubrum ezzemoulense* DSM 17463^T^/CECT 7099^T^ | NZ_NEDJ00000000 | NZ_NEDJ00000000 | NZ_NEDJ00000000 | NZ_NEDJ00000000 | NZ_NEDJ00000000 | AB663412 | NZ_NEDJ00000000 |
| *Halorubrum halophilum* B8^T^ | NZ_BBJP00000000 | NZ_BBJP00000000 | NZ_BBJP00000000 | NZ_BBJP00000000 | NZ_BBJP00000000 | EF077637 | NZ_BBJP00000000 |
| *‘Halorubrum hochstenium’* ATCC 700873 | NZ_AOJO00000000 | NZ_AOJO00000000 | NZ_AOJO00000000 | NZ_AOJO00000000 | NZ_AOJO00000000 | NZ_AOJO00000000 | NZ_AOJO00000000 |
| *Halorubrum kocurii*  JCM 14978^T^/BG-1^T^ | NZ_AOJH00000000 | NZ_AOJH00000000 | NZ_AOJH00000000 | NZ_AOJH00000000 | NZ_AOJH00000000 | AM900832 | NZ_AOJH00000000 |
| *Halorubrum lacusprofundi* ATCC 49239^T^/JCM 8891^T^ | HQ149375 | CP001365 | CP001365 | CP001365 | AB477181 | U17365 | CP001365 |
| *Halorubrum lipolyticum* JCM 13559^T^/DSM 21995^T^/9-3^T^ | **KM105851** | NZ_AOJG00000000 | **KM107969** | **KM107937** | AB477182 | DQ355814 | NZ_AOJG00000000 |
| *Halorubrum litoreum*  JCM 13561^T^/Fa-1^T^ | **KM105852** | **KM107923** | **KM107970** | **KM107938** | AB477183 | EF028067 | NZ_AOJF00000000 |
| *Halorubrum luteum* CECT 7303^T^/CGMCC 1.6783^T^/CGSA15^T^ | ND | KM980079 | ND | ND | AB820300 | DQ987877 | ND |
| *Halorubrum orientale* CECT 7145^T^/CGMCC 1.6295^T^/EJ-52^T^ | ND | KM980080 | ND | ND | AB820298 | AM235786 | ND |
| *Halorubrum persicum* C49^T^ | KJ152247 | KJ152276 | KJ152362 | **KM107947** | KJ152361 | HG421000 | NZ_NHOA00000000 |
| *Halorubrum rubrum* YC87^T^ | ND | KM980081 | ND | ND | KF286655 | JQ237124 | ND |
| *Halorubrum saccharovorum*  JCM 8865^T^/DSM 1137^T^ | HQ149376 | HQ149409 | **KM107971** | NZ_AOJE00000000 | AB477184 | U17364 | NZ_AOJE00000000 |
| *Halorubrum salinum* JCM 17093^T^/GX71^T^ | **LT578412** | **LT578413** | **LT578414** | ND | **LT578415** | HM063951 | ND |
| *Halorubrum sodomense* JCM 8880^T^/ATCC 33755^T^/RD 26^T^ | HQ149377 | HQ149410 | **KM107972** | **KM107939** | AB477185 | D13379 | NZ_FOYN00000000 |
| *Halorubrum tebenquichense*  JCM 12290^T^/DSM 14210^T^/CECT 5317^T^ | **KM105853** | **KM107924** | **KM107973** | **KM107940** | AB477186 | FR870448 | NZ_AOJD00000000 |
| *Halorubrum terrestre*  JCM 10247^T^/VKM B-1739T | **KM105854** | **KM107925** | **KM107974** | **KM107941** | AB477187 | AB090169 | NZ_AOIW00000000 |
| *Halorubrum tibetense* JCM 11889^T^/8W8^T^ | **KM105855** | **KM107926** | **KM107975** | **KM107942** | AB477188 | AY149598 | ND |
| *Halorubrum trapanicum* JCM 10477^T^/CBA1232 | **KM105856** | **KM107927** | **KM107976** | **KM107943** | AB477189 | AB663424 | NZ_AP017569 |
| *Halorubrum vacuolatum* JCM 9060^T^ | HQ149378 | HQ149411 | **KM107977** | **KM107944** | AB477190 | D87972 | NZ_FZNQ00000000 |
| *Halorubrum xinjiangense* JCM 12388^T^/BD-1^T^/CGMCC 1.3527^T^ | **KM105857** | **KM107928** | **KM107978** | **KM107945** | AB477191 | AY510707 | PRJNA303460 |
| *Halorubrum* sp. ARQ123^a^ | **LT578373** | **LT578383** | **LT578393** | ND | **LT578403** | **LT578361** | ND |
| *Halorubrum* sp. ASP57^a^ | **LT578369** | **LT578379** | **LT578389** | ND | **LT578399** | **LT578360** | ND |
| *Halorubrum* sp. C170^b^ | KJ152235 | KJ152278 | ND | KJ152412 | KJ152359 | **KT825566** | ND |
| *Halorubrum* sp. C191^b^ | **KM105859** | KJ152295 | **KM107980** | KJ152423 | KJ152348 | **HG420998** | NZ_NHNZ00000000 |
| *Halorubrum* sp. Cb34^b^ | KJ152237 | KJ152279 | KJ152363 | KJ152413 | KJ152360 | **HG421007** | NZ_NHPJ00000000 |
| *Halorubrum* sp. Ea1^b^ | KJ152244 | KJ152281 | **KM107981** | KJ152418 | KJ152338 | NZ_NHPG00000000 | NZ_NHPG00000000 |
| *Halorubrum* sp. Ea4p^b^ | KJ152242 | KJ152282 | KJ152380 | KJ152414 | KJ152344 | **HG421005** | ND |
| *Halorubrum* sp. Ea8^b^ | KJ152238 | KJ152283 | KJ152381 | KJ152415 | KJ152345 | **HG420997** | NZ_NHPF00000000 |
| *Halorubrum* sp. Ea10^b^ | KJ152241 | KJ152284 | KJ152376 | KJ152417 | KJ152337 | HQ425079 | ND |
| *Halorubrum* sp. Eb13^b^ | KJ152239 | KJ152280 | KJ152379 | KJ152420 | **KM107950** | **HG420999** | NZ_NHPE00000000 |
| *Halorubrum* sp. Ec5^b^ | **KM105860** | KJ152292 | KJ152367 | KJ152432 | **KM107951** | HQ425094 | ND |
| *Halorubrum* sp. Ec15^b^ | KM105861 | KJ152291 | KJ152367 | KJ152428 | **KM107952** | **HG421004** | NZ_NHPD00000000 |
| *Halorubrum* sp. Fa5^b^ | **KM105863** | KJ152296 | KJ152373 | KJ152424 | **KM107953** | HQ425100 | ND |
| *Halorubrum* sp. Fb21^b^ | **KM105865** | KJ152297 | KJ152370 | KJ152425 | **KM107954** | **HG420996** | NZ_NHPC00000000 |
| *Halorubrum* sp. Fc2^b^ | KJ152249 | KJ152293 | KJ152364 | KJ152430 | **KM107955** | HQ425106 | ND |
| *Halorubrum* sp. G37^b^ | **KM105866** | KJ152298 | KJ152368 | KJ152433 | **KM107956** | **HG421001** | NZ_NHPB00000000 |
| *Halorubrum* sp. Ga2p^b^ | **KM105867** | KJ152290 | KJ152371 | KJ152429 | KJ152347 | **HG421002** | NZ_NHPA00000000 |
| *Halorubrum* sp. Ga36^b^ | **KM105869** | KJ152294 | KJ152366 | KJ152431 | KJ152346 | **HG421003** | NZ_NHOZ00000000 |
| *Halorubrum* sp. Ga66^b^ | **KM105870** | KJ152289 | KJ152374 | KJ152426 | KJ152358 | HQ425110 | ND |
| *Halorubrum* sp. Hd13^b^ | KJ152243 | KJ152287 | KJ152378 | KJ152416 | **KM107958** | NZ_NHOY00000000 | NZ_NHOY00000000 |
| *Halorubrum* sp. Ib24^b^ | KJ152240 | KJ152285 | KJ152382 | KJ152421 | **KM107959** | **HG421006** | NZ_NHOX00000000 |
| *Halorubrum* sp. Ib25^b^ | KJ152245 | **KM107930** | KJ152375 | KJ152419 | **KM107960** | HQ425137 | ND |
| *Halorubrum* sp. Ib43^b^ | KJ152246 | KJ152286 | KJ152383 | KJ152422 | **KM107951** | HQ425139 | ND |
| *Halorubrum* sp. SD612^c^ | **LT578375** | **LT578385** | **LT578395** | **LT578410** | **LT578405** | **LT578363** | NZ_NEWI00000000 |
| *Halorubrum* sp. SD683^c^ | **LT578376** | **LT578386** | **LT578396** | **LT578411** | **LT578406** | **LT578362** | NZ_NEWJ00000000 |

^a^Strains isolated in this study from Isla Cristina solar saltern in Spain (37º 12’ 26” N; 7º 21’ 44” W).

^b^Strains isolated in this study from the lake Aran-Bidgol, Iran (34°31′ 25” N; 51° 53’ 40” E).

^c^Strains isolated in this study from the Walvis Bay solar saltern in Namibia (23º 1’ 27” S; 14º 26’ 57” E).

**Supplementary Table S2. Oligonucleotide primers used for PCR amplification and sequencing.** Primer names are derived from the locus they amplify. For the housekeeping genes, the primer name is followed by the position in the alignment that they prime, whether they were forward (F) or reverse (R) and whether they had M13 sequences used for sequencing (Fullmer et al., 2014; Ram-Mohan et al., 2014). *T*_A_ is the annealing temperature as determined by gradient PCR. M13 sequences are indicated in bold. *nd, not determined.

| **Primer** | ***T_A_* (ºC)** | **Sequence (5’-3’)** |
| --- | --- | --- |
| 16S_rRNA_ArchF | 50 | TTCCGGTTGATCCTGCCGGA |
| 16S_rRNA_ArchR | 50 | GGTTACCTTGTTACGACTT |
| 16S_rRNA_D34 | nd* | GGTCTCGCTCGTTGCCTG |
| 16S_rRNA_B36 | nd* | GGACTACCAGGGTATCTA |
| atpB_409F_M13(-21) | 60.0 | **TGTAAAACGACGGCCAGT**AACGGTGAGSCVATSAACCC |
| atpB_906R_M13R | 60.0 | **CAGGAAACAGCTATGAC**TTCAGGTCVGTRTACATGTA |
| EF-2_634F_M13(-21) | 61.0 | **TGTAAAACGACGGCCAGT**ATCCGCGCTBTAYAASTGG |
| EF-2_1147R_M13R | 61.0 | **CAGGAAACAGCTATGAC**TGGTCGATGGWYTCGAAHGG |
| glnA_329F_M13(-21)F | 69.6 | **TGTAAAACGACGGCCAGT**AGGTACGGGTTACASGACGG |
| glnA_860R_M13R | 69.6 | **CAGGAAACAGCTATGAC**CCTCGCSCCGAARGACCTCGC |
| ppsA_585F_M13(-21)F | 66.0 | **TGTAAAACGACGGCCAGT**CCGCGGTARCCVAGCATCGG |
| ppsA_1120R_M13R | 66.0 | **CAGGAAACAGCTATGAC**ATCGTCACCGACGARGGYGG |
| rpoB_962F_M13 | 63.7 | **TGTAAAACGACGGCCAGT**TCGAAGAGCCGGACGACATGG |
| rpoB_1504R_M13 | 63.7 | **CAGGAAACAGCTATGACC**GGTCAGCACCTGBACCGGNCC |

**Supplementary Table S3. Sequence information of individual housekeeping genes and five concatenated genes (*atpB*, *EF-2*, *glnA*, *ppsA*, and *rpoB’*). nt, number of nucleotides; IS, invariable sites; VS, variable but parsimony-uninformative sites; PI, parsimony-informative sites.**

| **Locus** | **nt** | **IS** | **VS** | **PI (%)** |
| --- | --- | --- | --- | --- |
| *atpB* | 496 | 344 | 49 | 103 (21) |
| *EF-2* | 507 | 304 | 51 | 152 (30) |
| *glnA* | 526 | 321 | 62 | 143 (27) |
| *ppsA* | 514 | 238 | 73 | 203 (39) |
| *rpoB’* | 522 | 320 | 72 | 130 (25) |
| Concatenated genes | 2565 | 1546 | 302 | 717 (28) |

**Supplementary Table S4. Evolutionary models used for constructing the phylogenies based on the 16S rRNA gene, individual and concatenated housekeeping genes, determined by means of jModelTest program.** TVM, transversional mode; I, invariable sites included; G, rate variation among sites taken into account; TIM, transitional model; GTR, general time-reversible model.

| **Locus** | **Best model** |
| --- | --- |
| 16S rRNA | TIM2 + I + G + F |
| *atpB* | TVM + I + G + F |
| *EF-2* | TVM + I + G + F |
| *glnA* | TIM1 + I + G + F |
| *ppsA* | GTR + I + G |
| *rpoB’* | GTR + I + G |
| Contatenated | GTR + I + G |
